# Prioritizing barriers and strategies for non-toxic material adoption in interior design

**DOI:** 10.3389/fpubh.2026.1901022

**Published:** 2026-09-04

**Authors:** Vigneshkumar Chellappa, Shalini Sharma, Martina Zelenakova, Jozef Selín

**Affiliations:** 1 School of Design, The Hong Kong Polytechnic University, Kowloon, Hong Kong SAR, China; 2 Amity School of Design, Amity University Noida, Noida, India; 3 Faculty of Civil Engineering, Technical University of Kosice, Kosice, Slovakia; 4 Department of Hydraulics and Hydro Informatics, Tashkent Institute of Irrigation and Agricultural Mechanization Engineers, National Research University, Tashkent, Uzbekistan

**Keywords:** environmental exposure, indoor air pollution, interior design, non-toxic materials, occupational health, sustainable materials

## Abstract

**Background:**

Growing concern about indoor environmental quality and occupant health has underscored the need to select interior materials that minimize harmful chemical emissions and reduce exposure to toxic substances. However, despite growing awareness of healthier interior environments, the adoption of non-toxic materials remains limited due to technical, informational, economic, and organizational challenges.

**Methods:**

This study adopted a three-stage multi-method approach. First, a Comprehensive Literature Review (CLR) was conducted to identify 19 barriers and 15 strategies related to the adoption of non-toxic materials in interior design. Second, a two-round Delphi technique was used to validate and refine these factors, involving 22 expert panelists in Round 1 and 16 in Round 2. Third, the Technique for Order Preference by Similarity to Ideal Solution (TOPSIS), a multi-attribute decision-making method, was used to prioritize the identified barriers and strategies based on expert qualification-weighted scores.

**Results:**

The TOPSIS analysis identified the five most critical barriers as lack of awareness (Rank 1), limited client interest (Rank 2), cultural resistance (Rank 3), lack of collaboration (Rank 4), and limited data availability (Rank 5). The five highest-priority strategies were educational campaigns (Rank 1), client education initiatives (Rank 2), fostering a collaborative design culture (Rank 3), interdisciplinary collaboration (Rank 4), and developing comprehensive resource databases (Rank 5). These findings indicate that the adoption of non-toxic materials is mainly constrained by informational, cultural, and demand-side barriers, while educational and collaborative interventions offer the most effective responses.

**Conclusion:**

The study provides a structured, evidence-based framework for understanding and prioritizing barriers and strategies for adopting non-toxic materials in interior design. The findings offer practical guidance for designers, policymakers, and industry stakeholders seeking to promote healthier interior environments through informed material selection and coordinated action.

## 1 Introduction

Interior design plays a critical role in shaping the quality, safety, and experience of indoor environments. Beyond aesthetics and functionality, the materials used in interior spaces directly influence indoor air quality, occupant comfort, and human health (1). In contemporary built environments, individuals spend a substantial portion of their daily lives indoors, which increases the importance of selecting materials that do not release harmful substances into the surrounding environment. Many conventional interior materials, including paints, adhesives, coatings, flooring products, composite wood, and synthetic finishes, may contain chemicals that contribute to indoor air pollution and pose risks to occupants over time (2, 3). As awareness of healthy buildings continues to grow, material toxicity has become an increasingly important concern in interior design practice and research. The growing emphasis on sustainable development has encouraged designers and construction professionals to rethink how material choices affect both environmental performance and human wellbeing. While sustainable design has often focused on energy efficiency, waste reduction, and resource conservation (4), there is now greater recognition that sustainability must also include the health implications of materials used inside buildings. In this context, non-toxic materials are gaining importance because they offer the potential to reduce harmful emissions, improve indoor environmental quality, and support healthier living and working spaces (5, 6). Their use is particularly relevant in interior design, where direct human interaction with surfaces, finishes, and furnishings is constant and prolonged.

Despite these benefits, the adoption of non-toxic materials in interior design remains limited in practice. In many projects, material selection continues to be guided primarily by cost, availability, appearance, and conventional performance criteria, while health-related considerations receive less attention (7, 8). Designers may face difficulties in identifying safe alternatives, evaluating product claims, or convincing clients to prioritize healthier options (9). Similarly, insufficient technical knowledge, a lack of collaboration among project stakeholders, and weak market demand may further slow the transition to non-toxic materials in the built environment (10, 11). These challenges suggest that the problem is not only technical but also organizational, informational, and behavioral (12). This issue is particularly important in developing regions, where rapid urbanization, expanding construction activities, limited regulatory enforcement, and cost-sensitive project environments often make the adoption of healthier materials more difficult (13–15). In such contexts, immediate economic and functional considerations frequently take precedence over long-term health and environmental concerns. India represents a particularly significant case in this regard. As one of the fastest-growing construction and interior design markets, India is experiencing a substantial increase in residential, commercial, and institutional interior development. At the same time, concerns about indoor air quality, material safety, and public health are becoming increasingly relevant due to dense urban living, rising exposure to synthetic building materials, and growing awareness of healthy built environments (16, 17). However, the use of non-toxic materials in Indian interior design practice remains insufficiently studied, despite the country’s scale, diversity, and growing design sector.

Existing studies have examined sustainable materials, healthy buildings, and environmentally responsible design practices (16, 18). Although some studies have addressed non-toxic materials, their focus has generally been on the broader built environment rather than specifically on interior design spaces (19, 20). Moreover, strategies for overcoming barriers to adopting non-toxic materials in interior design remain underexplored. This gap is especially significant in the Indian context, where local market conditions, professional practices, client awareness, and regulatory frameworks may shape material decisions differently from those in developed countries. Therefore, there is a need for a systematic framework that can identify the most critical barriers and prioritize practical strategies. Without such analysis, efforts to promote non-toxic materials may remain fragmented and less effective. Accordingly, this study focuses on India to examine barriers to adopting non-toxic materials in interior design and to identify enabling strategies to support healthier, more informed design practices. To achieve this, the study employed a combined methodological approach comprising a literature review, the Delphi technique, and the Technique for Order Preference by Similarity to Ideal Solution (TOPSIS). Through this approach, the study aimed to provide a structured, evidence-based understanding of the factors hindering the use of non-toxic materials and the actions that can most effectively encourage their wider adoption. The novelty of this work lies in its application of a multi-attribute decision-making framework to a previously underexplored intersection of material health, interior design practice, and the Indian built environment context, thereby providing actionable, priority-ordered guidance for designers and stakeholders. By identifying the most influential barriers and the most effective strategies, the study supports efforts to create interior spaces that are not only visually and functionally successful but also safer and healthier for occupants in India.

## 2 Literature review

Interior spaces consist of multiple material layers, including finishes, furnishings, adhesives, sealants, coatings, and engineered wood products, many of which may contain volatile organic compounds, formaldehyde, phthalates, flame retardants, and other hazardous substances. These emissions can reduce indoor air quality and adversely affect occupant health and well-being (21, 22). Since people spend most of their time indoors, the health implications of interior materials have become an important concern in design, architecture, and environmental health research. In response, non-toxic materials have gained increasing attention as a preventive approach to creating healthier built environments. These materials are generally understood to minimize harmful chemical emissions, reduce indoor pollution, and pose fewer health risks during installation, use, and disposal (21, 23). Although they are often discussed within the broader concept of sustainable design, non-toxic materials specifically address material health and human exposure. Unlike sustainable materials, which are commonly assessed through energy use, recyclability, and environmental impact, non-toxic materials are particularly valued for their contribution to healthier indoor environments (24). Therefore, material selection in interior design should not rely only on aesthetics, cost, and performance, but also on its effects on indoor environmental quality, user comfort, and long-term human health across the building lifecycle.

Despite this growing recognition, the literature indicates that the adoption of non-toxic materials in interior design remains limited and inconsistent. One major reason is the lack of awareness among both designers and clients regarding the health risks associated with conventional materials and the benefits of safer alternatives (25). Several studies on sustainable interior design have shown that professionals often support the idea of healthier, more sustainable material selection in principle, yet its implementation remains constrained by limited knowledge, insufficient training, and difficulty identifying appropriate products (25, 26). In many cases, clients are also unfamiliar with the concept of non-toxic materials or do not consider material toxicity to be an important criterion in project decision-making (26). This gap between awareness and practice has been widely recognized as a central challenge in advancing healthier interior environments. Another recurring theme in the literature is the influence of client preferences and market demand on material selection. Interior designers frequently operate within client-driven projects, where budget, aesthetics, deadlines, and perceived value shape choices. Even when designers are personally committed to using healthier materials, they may be unable to prioritize them if clients are unwilling to support such decisions. Research in sustainable design has repeatedly shown that client reluctance, low demand, and concern over additional costs can significantly reduce the likelihood of adopting alternative materials (26, 27). In this context, non-toxic materials may be viewed as desirable but non-essential, particularly when their benefits are not immediately visible or well understood. This suggests that client education and demand creation are critical to shifting material practices in the industry (25, 27). The literature also highlights the role of cultural and professional resistance in slowing the transition toward healthier materials. Interior design practice is often influenced by established habits, familiar products, preferred suppliers, and conventional assumptions about what constitutes quality, beauty, and performance. Designers and contractors may continue to use traditional materials because they are trusted, readily available, and well understood in terms of application and maintenance. By contrast, non-toxic alternatives may be perceived as unfamiliar, expensive, less aesthetically flexible, or riskier to specify (25). Such perceptions can discourage experimentation and reinforce dependence on conventional products. Studies on sustainability transitions in the built environment suggest that this form of resistance is not merely individual but embedded within broader professional cultures and industry norms (25, 28). In addition, aesthetic expectations and performance concerns may affect adoption, as non-toxic materials may be perceived as unable to fully satisfy design preferences, durability requirements, or technical expectations compared with conventional materials (25, 29, 30).

Access to reliable information has also been identified as a major challenge in the literature. Designers often face difficulty obtaining clear and comparable data on material ingredients, chemical emissions, certifications, and long-term performance (23, 31). Product claims may be inconsistent, incomplete, or overly promotional, making it difficult to distinguish genuinely non-toxic materials from those marketed as environmentally friendly. This issue is closely related to concerns about greenwashing, in which misleading or vague sustainability claims create confusion and erode trust (32). As a result, the absence of transparent and credible product information can discourage designers from selecting healthier materials, even when they are motivated to do so. The literature, therefore, emphasizes the importance of material databases, third-party certifications, and standardized disclosure frameworks in supporting informed specification (21, 23). At the same time, uncertainty may also arise from inadequate testing and certification systems, inconsistent standards, and concerns about hidden health risks, all of which complicate professional decision-making and reduce confidence in material claims (21, 22, 32). Collaboration among stakeholders is another important issue discussed in previous studies. The selection of interior materials is rarely determined by designers alone; rather, it involves interaction among clients, suppliers, contractors, architects, consultants, and project managers. Where communication is fragmented and responsibilities are unclear, health-related material considerations may be overlooked (25, 27). Research on integrated design and sustainable construction has shown that interdisciplinary collaboration is essential for aligning project goals, sharing technical knowledge, and resolving practical constraints. In the context of non-toxic materials, effective collaboration can improve access to information, support better procurement decisions, and ensure that healthier material choices are considered early in the design process rather than being introduced too late to be feasible. However, a lack of collaboration, limited availability of sources, complex supply chains, and time constraints can significantly slow adoption, particularly when projects operate under tight schedules and market limitations (9, 27, 33). The literature further indicates that regulatory and institutional support for adopting non-toxic materials is often inadequate. Although green building frameworks and sustainability standards have helped increase awareness of environmental performance, they do not always provide sufficient emphasis on material health and chemical safety. In many regions, regulations governing interior product emissions, ingredient disclosure, or healthy material certification remain limited, inconsistent, or fragmented (21, 32). This creates a context in which designers may lack both external incentives and practical guidance for prioritizing non-toxic materials. In addition, limited research and development, shortage of skilled professionals, and knowledge gaps regarding lifecycle impacts can further constrain the effective adoption of healthier materials (23, 24, 27, 34). Scholars have therefore argued that stronger standards, policy support, and professional guidelines are needed to encourage wider adoption and to reduce the burden currently placed on individual practitioners to evaluate product safety independently.

Several studies have proposed strategies to overcome these barriers. Education and training are among the most frequently recommended interventions, as they can improve designers’ knowledge, build confidence in evaluating safer materials, and increase client awareness of health-related issues (25, 26). Client education initiatives are particularly important in projects where cost and visual appeal tend to dominate decision-making, as they help communicate the long-term value of healthier materials (25, 27). Resource databases and material libraries have also been identified as useful tools for improving access to product information and reducing the time required for evaluation (23, 25). Similarly, stronger cross-disciplinary collaboration can help integrate material health into project workflows and support better decision-making (25, 27). Some researchers also emphasize the importance of advocacy, cultural change, supplier networks, certification mandates, third-party testing, and streamlined procurement and decision-making processes in normalizing the use of healthier materials within professional practice (9, 28, 31, 32) (Niero et al., 2021). In addition, life cycle cost analysis, research and development investment, indoor air quality monitoring, and reuse-oriented practices have been suggested as supporting strategies for strengthening both confidence and feasibility in non-toxic material adoption (21, 23, 30, 34).

## 3 Methodology

Three main efforts were undertaken to achieve the study’s objectives. First, the barriers and strategies for using non-toxic materials were identified through a comprehensive literature review (CLR). Second, a Delphi survey was conducted with professionals to validate and evaluate the barriers and strategies identified from the CLR. Third, following the Delphi survey, a multi-attribute decision-making (MADM) method was used to prioritize the barriers and strategies based on the professionals’ ratings. The following sections discuss each of these steps in detail.

### 3.1 Identification of barriers and strategies

The barriers and strategies were identified based on a CLR. To locate relevant literature, keywords such as non-toxic materials, hazardous building materials, sustainable built environment, healthy building materials, green buildings, material selection, sustainable materials, sustainable construction, and interior design practices were used in databases such as Scopus and Web of Science (WoS). These keywords were selected to capture studies addressing health-conscious material selection, sustainability in interior environments, and the challenges of reducing hazardous material use in design practice. The selected databases were used for their broad coverage of peer-reviewed research (35) and recognized reliability for systematic reviews in built environment and design-related studies (36, 37). The initial search yielded 118 documents, comprising 66 records from Scopus and 52 from WoS. The initial search results were filtered using predefined inclusion criteria, including English-language publications, a publication period of 2011–2025, journal articles, and studies relevant to sustainable materials, the built environment, and interior design. Following a thorough review of the retrieved articles, 53 were selected as eligible and underwent in-depth analysis. After removing irrelevant and duplicate records (*n* = 12), the remaining studies were screened by title, abstract, and full text to assess their relevance to the objectives of the present study. Only studies that explicitly discussed barriers, challenges, enabling factors, recommendations, or strategies related to the adoption of non-toxic or less-hazardous materials in built environments and interior spaces were retained for detailed review. Based on this process, the final set of selected papers (*n* = 16) was subsequently retrieved for further analysis. Each paper was thoroughly reviewed independently by two researchers. During this process, the researchers manually identified the barriers and strategies reported in each study and recorded them in an Excel sheet together with their corresponding source references. This independent extraction process was carried out to improve the reliability and consistency of the findings. After completing the individual reviews, the two researchers compared their extracted items, cross-checked the identified barriers and strategies, and discussed any differences in interpretation. Any inconsistencies were resolved through mutual discussion and verification against the original articles until agreement was reached. This process ensured that the final list of barriers and strategies was accurate, consistent, and clearly traceable to the supporting literature. Table 1 presents the barriers and strategies identified through the CLR. In total, 19 barriers and 15 strategies were extracted from the selected studies. This process formed the foundation for the expert validation and prioritization conducted in the subsequent stages of the study.

**Table 1:** Barriers and strategies identified from the literature.

Code	Item	Description	Source
Barriers			
B1	Aesthetic Limitations	Non-toxic materials may not meet design expectations or preferences, leading to reluctance to use them.	Ashour et al. (25)
B2	Complex Supply Chains	Complications in the supply chain for non-toxic materials can lead to delays and uncertainties in availability.	Payer et al. (9) and Häkkinen and Belloni (27)
B3	Cultural Resistance	Resistance to changing established practices and preferences for traditional materials.	Kanters (28) and Ashour et al. (25)
B4	Greenwashing Issues	The prevalence of misleading claims about sustainability can create skepticism regarding the effectiveness of non-toxic options.	Li et al. (32)
B5	Hidden Health Risks	Some non-toxic materials may pose undisclosed health risks, complicating designers’ decisions.	Liu et al. (21) and Rochikashvili and Bongaerts (22)
B6	Higher Initial Costs	Non-toxic materials typically have higher upfront costs than conventional options, which can affect project budgets.	Akadiri (33) and Häkkinen and Belloni (27)
B7	Inadequate Testing and Certification	Lack of standardized testing and certification creates uncertainty about the quality and safety of non-toxic materials.	Liu et al. (21) and Li et al. (32)
B8	Inconsistent Standards	The absence of uniform standards creates confusion about the quality of non-toxic materials.	Liu et al. (21)
B9	Insufficient Research and Development	Limited investment in R&D slows the innovation and availability of non-toxic materials.	Häkkinen and Belloni (27) and Kappenthuler and Seeger (34)
B10	Knowledge Gaps Regarding Lifecycle Impact	A lack of understanding about the lifecycle impacts of non-toxic materials complicates decision-making.	Pollini and Rognoli (23) and Massimino and Sciuto (24)
B11	Lack of Awareness	Insufficient knowledge among designers, builders, and clients regarding the benefits of non-toxic materials.	Ashour et al. (25), and Mohsen and Matarneh (26)
B12	Lack of Collaboration	Insufficient collaboration among stakeholders can limit the effective use of non-toxic materials.	Ashour et al. (25) and Häkkinen and Belloni (27)
B13	Limited Availability of Sources	Challenges in sourcing a variety of non-toxic materials, especially in certain regions or markets.	Akadiri (33) and Mohsen and Matarneh (26)
B14	Limited Client Interest	Clients often prioritize cost and aesthetics over sustainability, hindering the adoption of non-toxic materials.	Häkkinen and Belloni (27) and Mohsen and Matarneh (26)
B15	Limited Data Availability	Difficulty accessing data and research on non-toxic materials can impede their adoption.	Lamé et al. (31), and Pollini, and Rognoli (23)
B16	Performance Concerns	Concerns regarding the durability and performance of non-toxic materials compared to traditional options.	Florez and Castro-Lacouture (29) and Shaharuzaman et al. (30)
B17	Regulatory Hurdles	Complex regulations and building codes may favor traditional materials, complicating the use of non-toxic materials.	Ashour et al. (25) and Li et al. (32)
B18	Shortage of Skills	A lack of trained professionals knowledgeable in non-toxic materials limits their effective application.	Ashour et al. (25) and Mohsen and Matarneh (26)
B19	Time Constraints	Tight project timelines may deter careful consideration and sourcing of non-toxic materials.	Ashour et al. (25) and Häkkinen and Belloni (27)
Strategies			
S1	Conduct Life Cycle Cost Analysis	Demonstrate long-term savings associated with non-toxic materials over time.	Pollini and Rognoli (23) and Shaharuzaman et al. (30)
S2	Develop Client Education Initiatives	Create personalized presentations for clients to demonstrate the benefits of non-toxic materials in designs.	Häkkinen and Belloni (27) and Ashour et al. (25)
S3	Develop Comprehensive Resource Databases	Create accessible databases that compile information and performance metrics on non-toxic materials.	Pollini and Rognoli (23) and Ashour et al. (25)
S4	Encourage Cultural Shift	Initiate social campaigns to positively change perceptions towards sustainable and non-toxic materials.	Kanters (28) and Ashour et al. (25)
S5	Establish a Supplier Network	Partner with local suppliers to improve accessibility and reduce costs for non-toxic materials.	Payer et al. (9) and Häkkinen and Belloni (27)
S6	Establish IAQ Monitoring Protocols	Set up protocols to monitor indoor air quality and ensure that materials used do not contribute to poor air quality.	Liu et al. (21)
S7	Foster a Collaborative Design Culture	Encourage cross-sector collaboration among the design, construction, and supply chain sectors to promote non-toxic options.	Ashour et al. (25) and Häkkinen and Belloni (27)
S8	Foster Interdisciplinary Collaboration	Form cross-disciplinary teams for knowledge sharing to enhance the understanding of non-toxic materials.	Ashour et al. (25)
S9	Implement Certification Mandates	Require that all materials used in projects meet established safety and sustainability certifications.	Liu et al. (21) and Li et al. (32)
S10	Implement Reuse and Upcycling Programs	Encourage the reuse of existing materials and upcycling to reduce waste and promote sustainable practices.	Payer et al. (9) and Kanters (28)
S11	Invest in Educational Campaigns	Organize workshops, presentations, and social campaigns to improve awareness and knowledge about non-toxic materials.	Ashour et al. (25) and Mohsen and Matarneh (26)
S12	Invest in R&D Initiatives	Support innovation by collaborating with research institutions to develop better non-toxic options.	Kappenthuler and Seeger (34) and Häkkinen and Belloni (27)
S13	Promote Third-Party Testing and Certification	Encourage independent testing and certification for non-toxic materials to build trust.	Liu et al. (21) and Li et al. (32)
S14	Provide Streamlined Decision-Making Processes	Create checklists and frameworks that simplify the selection of non-toxic materials.	Lamé et al. (31) and Niero et al. (2021)
S15	Streamline Procurement Processes	Improve sourcing practices to enhance the timeline for access to non-toxic materials.	Payer et al. (9) and Häkkinen and Belloni (27)

### 3.2 Delphi survey

#### 3.2.1 Participant identification

After the barriers and strategies had been identified, the subsequent phase focused on confirming their validity and establishing their order of importance through the Delphi technique. This method was adopted because it is well-suited to obtaining informed judgments from experts on a defined subject and to developing consensus regarding the relative significance of different factors (38). As noted by Chellappa et al. (39), the effectiveness of a Delphi study depends greatly on the knowledge, experience, and overall composition of the expert panel, making participant selection a critical step in the research design. Accordingly, the present study adopted a structured three-step procedure to identify and recruit suitable panel members. Consistent with the recommendation of Karakhan and Al-Bayati (40), the Delphi panel comprised industry practitioners to ensure that the responses reflected practical knowledge, industry relevance, and professional experience. In the initial stage, the researchers applied their professional judgment to assess the suitability of potential participants. Key criteria, including job role and educational qualifications, further guided expert identification. Through this process, 40 practitioners were identified as appropriate candidates, all of whom were involved in projects located in New Delhi and the National Capital Region (NCR), particularly Gurgaon and Noida, India. These locations were chosen because they are among the most significant and rapidly expanding construction centers in Northern India, characterized by intensive residential and commercial development, increasing environmental concerns, and a growing emphasis on healthier interior environments, thereby providing a highly relevant setting for investigating the adoption of non-toxic materials. The identified experts were then contacted, informed of the research purpose and objectives, and formally invited to participate in the Delphi study. Of the 40 experts approached, 24 agreed to participate and were included in the study.

An email was subsequently distributed to the 24 individuals who expressed willingness to participate, requesting details regarding their professional experience, academic qualifications, and other relevant credentials. The collected information was used to evaluate their appropriateness for inclusion in the expert panel. To assess their eligibility, a point-based qualification system (see Table 2) was used, in which participants were evaluated on criteria including educational attainment, years of industry experience, research publications, committee involvement, and professional registration. The combined score across these criteria was used to determine whether an individual could be considered an expert. In accordance with the criteria adopted in earlier studies (38, 39), participants who scored 11 or higher were classified as experts. This scoring approach has been widely recognized and applied in construction management research (40). Out of the 24 individuals contacted, 22 submitted the requested information for qualification assessment. After evaluating their responses, all 22 participants exceeded the minimum threshold of 11 points and were therefore confirmed as qualified experts. The final panel of 22 experts falls within the panel size recommended in prior Delphi-based studies (39, 41).

**Table 2:** Expert qualification criteria.

Criteria	Weight
Experience in years	1 point each
Educational qualification	Bachelor’s degree (4 points)
	Master’s degree (+2 points)
	PhD (+2 points)
Certifications/credentials	1 point (each)
Professional registration	3 points each
Publications (in interior design-allied domain)	Books (4 points each)
	Book chapters (2 points each)
	Journal (2 points each)
	Conference proceedings (0.5 point each)

#### 3.2.2 Questionnaire design and data collection

A structured questionnaire was designed, based on the barriers and strategies identified in the literature review, to assess their significance through expert judgment. The instrument consisted of two main sections. The first section asked respondents to evaluate the extent to which each identified barrier influences the adoption of non-toxic materials in interior design spaces. The second section focused on the proposed strategies and required respondents to assess the relative importance of each in overcoming those barriers. For the barriers section, an example item is: “Limited awareness of non-toxic material alternatives among designers and clients significantly hinders the adoption of non-toxic materials in interior design spaces.” For the strategies section, an example item is: “Developing educational initiatives for clients is an effective strategy for increasing the adoption of non-toxic materials in interior design spaces.” Participants were asked to indicate their responses using a five-point Likert scale. In the barriers section, the scale ranged from 1 = strongly disagree to 5 = strongly agree, reflecting the perceived level of influence of each barrier. A similar rating approach was applied to the strategies section to evaluate their importance.

Before administering the questionnaire to the Delphi panel, a pilot study was conducted with four interior designers, each with at least 3 years of professional experience, who were not included in the main expert panel. The purpose of this pilot phase was to examine the clarity, readability, and appropriateness of the questionnaire items. Feedback from the pilot participants was used to refine the instrument’s wording and ensure the questions were easily understood and interpreted consistently. Following the pilot testing and necessary revisions, the final questionnaire was distributed to the expert panel for evaluation and feedback. The survey was administered through a web-based platform, allowing participants to complete it within a defined response period. At the end of the data collection period, the survey was closed automatically. Throughout the study, the panelists’ anonymity was maintained, meaning participants were unaware of one another’s identities or responses. The research team independently coordinated the entire process to ensure confidentiality and reduce the possibility of bias. The Delphi study was conducted in two rounds. In the first round, the panelists were presented with the identified barriers and strategies and asked to review and validate their relevance to interior design practice. In the second round, the experts were asked to evaluate and rate the influence of the validated barriers and strategies to determine their relative importance.

#### 3.2.3 Determining consensus

**Figure 1:**
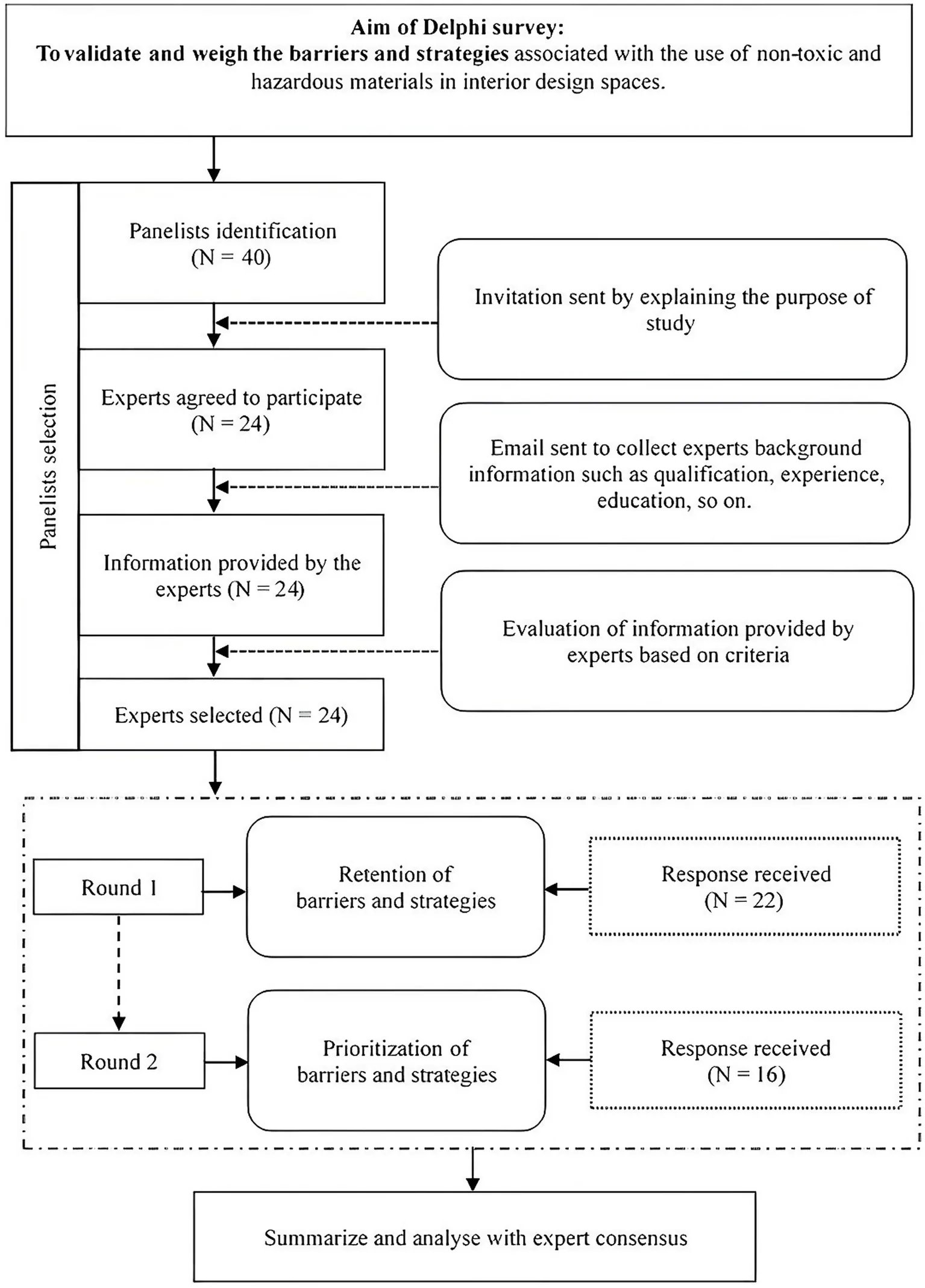
Delphi method flow chart.

Karakhan et al. (42) note that the strength of the Delphi technique lies in its structured group process, which is specifically designed to build consensus among experts. By encouraging the convergence of expert opinions and promoting strong agreement, the Delphi method helps reduce individual bias and enhance the credibility and robustness of the findings (38). To determine whether consensus has been reached, researchers commonly rely on statistical indicators that measure the extent of agreement among panel members and highlight areas of both consistency and variation in their responses. For studies involving relatively small sample sizes, previous research has identified the standard deviation (SD) together with the 50% majority rule as reliable measures for evaluating consensus (39, 42). Following the approach adopted in earlier studies, an SD value of 1.64 or below was taken to indicate an acceptable level of agreement among the experts (40). While some studies in construction management advocate for a stricter SD threshold (e.g., SD < 1.00) or employ alternative measures such as the Content Validity Ratio (CVR) or Kendall’s W, an SD of ≤ 1.64 on a 5-point Likert scale is theoretically justified in exploratory studies involving diverse professional panels. In such contexts, a slightly broader SD accommodates legitimate interdisciplinary variations in perspective—such as differences in emphasis between interior designers, architects, and sustainability consultants—while still indicating a central tendency of agreement. This threshold is consistent with established practice in construction management and decision science research (39, 40, 43). Crucially, this study did not rely on the SD threshold in isolation; it was strictly paired with the 50% majority rule, so that consensus was confirmed only when both conditions were satisfied simultaneously. This dual-criterion approach provides a more robust and conservative basis for establishing consensus than either measure applied independently (42, 43). Accordingly, responses were considered to have reached consensus when their SD did not exceed this threshold. In addition, the 50% majority rule was applied, meaning that consensus was further supported when more than half of the panelists agreed or disagreed on a given item (43, 44). In the present study, both of these criteria were used jointly to confirm that the level of agreement among the experts reflected a meaningful and dependable consensus. Figure 1 presents the flowchart of the Delphi technique used in this study.

### 3.3 Multi-attribute decision-making method

MADM methods are used when several alternatives must be assessed against multiple criteria to identify the most preferred option. In the present study, TOPSIS, originally proposed by Hwang and Yoon (45), was employed to evaluate the overall performance of the identified barriers and strategies using Delphi survey scores. TOPSIS is one of the most frequently applied MADM techniques because it provides a systematic way to rank alternatives based on their closeness to the ideal solution and their distance from the worst-case solution. In essence, the method assumes that the best alternative should be closest to the positive ideal solution and farthest from the negative ideal solution. This method was considered appropriate for the current study because it allows multiple barriers and strategies to be assessed simultaneously within a clear and structured analytical framework. By comparing each item against an ideal reference point, TOPSIS enables identification of the barriers with the greatest influence and the strategies with the highest priority for implementation, thereby supporting more effective decision-making for sustainable interior design practice. The usefulness of TOPSIS in addressing MADM problems has been demonstrated in a range of construction management studies (46, 47, 53). The TOPSIS procedure in this study followed a series of standard analytical steps (47). First, a decision matrix (A) was developed using the expert ratings collected through the Delphi survey. As noted earlier, each barrier and strategy was rated on a five-point Likert scale, with values ranging from 1 (strongly disagree) to 5 (strongly agree). Since the criteria were measured on the same response scale yet still needed to be comparable for ranking purposes, the next step was to normalize the values in the decision matrix. This normalization process transformed the raw ratings into dimensionless values, thereby enabling a consistent and unbiased comparison of all criteria included in the analysis. The normalized value r_{ij} is calculated using a sum-based normalization technique as follows:

$$\;r_{\mathrm{ij}}=\frac{x_{\mathrm{ij}}}{\sqrt{\sum_{i=1}^{m}x_{\mathrm{ij}}^{2}}}i=1,2,\ldots ,m;j=1,2,\ldots ,n$$ (1)

where *j* = number of alternatives; *i* = number of criteria; and $x_{\mathrm{ij}}$ = value of the *j* alternative for the *i* criterion.

Following normalization, the weighted-normalized decision matrix can be calculated as:

$$v_{\mathrm{ij}}=w_{j}\times r_{\mathrm{ij}}$$ (2)

where $w_{j}$ is the weight of the criteria for all *j*.

The next step is to calculate the ideal best ( $A^{+}$ ) and ideal worst ( $A^{-}$ ) solutions as follows:

$$A^{+}=\{v_{1}^{+},\ldots ,v_{n}^{+}\}=\{(\max_{j}v_{\mathrm{ij}}|i\in I^{\prime }\},\{(\min_{j}v_{\mathrm{ij}}|i\in I^{\prime \prime }\}$$ (3)

$$A^{-}=\{v_{1}^{-},\ldots ,v_{n}^{-}\}=\{(\min_{j}v_{\mathrm{ij}}|i\in I^{\prime }\},\{(\max_{j}v_{\mathrm{ij}}|i\in I^{\prime \prime }\}$$ (4)

where $I^{\prime }$ is associated with benefit criteria and $I^{\prime \prime }$ is associated with cost criteria. The benefit attribute is higher the better, and the cost attribute is lower the better.

The distance from each scheme to the positive ideal point ( $D_{i}^{+}$ ) and the negative ideal point ( $D_{i}^{-}$ ) is calculated to determine its relative position. The following formula can calculate n-dimensional Euclidean distance:

$$D_{i}^{+}=\sqrt{\sum_{j=1}^{n}{\left(v_{\mathrm{ij}}-v_{j}^{+}\right)}^{2}},i=1,2,3,\ldots ,m$$ (5)

$$D_{i}^{-}=\sqrt{\sum_{j=1}^{n}{\left(v_{\mathrm{ij}}-v_{j}^{-}\right)}^{2}},i=1,2,3,\ldots ,m$$ (6)

Here, $D_{i}^{+}$ and $D_{i}^{-}$ represent the distances from the positive and negative ideal solutions, respectively.

The relative closeness of each alternative to the ideal solution is determined as follows:

$$C_{i}^{*}=\frac{D_{i}^{-}}{\left(D_{i}^{+}+D_{i}^{-}\right)}$$ (7)

Where 0 ≤ $C_{i}^{*}$ ≤ 1. Finally, the alternatives are prioritized by their proximity to the ideal solution, with the closest ranked highest.

## 4 Results

### 4.1 Delphi findings

Table 3 presents the demographic profile and qualification assessment of the experts who participated in the Delphi study. As noted earlier, all 22 individuals satisfied the eligibility criteria and were therefore included in the expert panel. All 22 experts participated in Round 1, while 16 experts continued to Round 2. These 16 participants formed the final panel for the subsequent consensus assessment and prioritization analysis. The panel reflected a broad and diverse range of professional roles within the design and built environment sector. The participants included interior designers, designers, architects, consultants, workplace designers, a space designer, a founder, an interior design manager, a principal designer, a design manager, and a design director. This diversity ensured that the study captured insights from professionals occupying different functions and levels of responsibility across the industry. In terms of professional experience, the panel comprised experts with 2 to 21 years of industry experience, representing a balanced combination of early-career, mid-career, and highly experienced practitioners. Many participants had considerable professional experience, with several reporting more than 10 years and a few reporting nearly 20 years in the field. The panel’s average professional experience was 10.99 years, indicating a strong overall level of expertise. Regarding educational background, all participants held at least a bachelor’s degree, and a substantial proportion had also obtained a master’s degree, further enhancing the panel’s academic and professional strength. In addition, several experts reported holding professional certifications, professional registrations, and research-related contributions, demonstrating that the panel comprised individuals with both practical industry knowledge and professional credibility.

**Table 3:** Experts’ panel qualification data.

Experts participated	Participated in Delphi survey rounds	Role	Experience in years	Educational qualification		Certification	Professional registration	Publications	Total points	Weightage
				Bachelors’ degree	Masters’ degree			Conference proceedings		
1	Round 1	Interior Designer	8	4	0	0	3	0	15	Not applicable
2		Consultant	11	4	0	0	0	0	15	
3		Space Designer	12	4	2	1	0	0	19	
4		Architect	9	4	0	0	0	0	13	
5		Founder	5	4	2	0	0	1	12	
6		Interior Design Manager	7	4	0	1	0	0	12	
7	Rounds 1 & 2	Designer	4	4	2	1	3	0	14	0.04
8		Designer	11	4	0	1	3	0.5	19.5	0.06
9		Consultant	17	4	0	0	0	0	21	0.07
10		Senior Interior Designer	21	4	0	1	3	0	29	0.09
11		Architect	5	4	2	1	3	0	15	0.05
12		Interior Designer	14	4	2	1	3	0	24	0.08
13		Interior Designer	5	4	2	1	3	0	15	0.05
14		Interior Designer	19	4	0	0	0	0	23	0.07
15		Workplace Designer	9	4	2	1	0	0	16	0.05
16		Architect	6	4	0	1	3	0	14	0.04
17		Principal Designer	7	4	0	0	0	1	12	0.04
18		Designer	2	4	2	1	3	0	12	0.04
19		Designer	21	4	2	1	3	0	31	0.10
20		Designer	10	4	2	1	3	0.5	20.5	0.07
21		Design Manager	17	4	0	0	0	0	21	0.07
22		Design Director	20	4	0	1	3	0	28	0.09

#### 4.1.1 Round 1

In the first Delphi round, the barriers and strategies identified from the literature were presented to the expert panel for evaluation. The panelists were asked to review each item and determine whether it should be retained as relevant to the adoption of non-toxic materials in interior design. They were also invited to suggest any additional barriers or strategies that may have been overlooked. However, no new items were proposed, indicating that the experts considered the literature-derived list sufficiently comprehensive. As shown in Table 4, the majority of panelists supported retaining all identified barriers and strategies. Each item exceeded the 60% agreement threshold adopted for consensus, which is consistent with previous Delphi studies (43, 44). These findings demonstrate a satisfactory level of agreement among the panel members in Round 1 and confirm the relevance of the identified factors for further evaluation in the subsequent round. Among the barriers, limited client interest emerged as the most strongly supported factor influencing the adoption of non-toxic materials, receiving unanimous agreement from the panel. Among the strategies, several items also achieved unanimous retention, including conducting life cycle cost analysis, developing client education initiatives, establishing a supplier network, fostering a collaborative design culture, implementing reuse and upcycling programs, investing in R&D, promoting third-party testing and certification, and streamlining procurement processes. The strong agreement on these strategies suggests that the experts viewed them as particularly important measures for supporting the wider use of non-toxic materials in interior design practice. During this round, panelists were also invited to explain their reasons for not retaining certain items. For example, some experts indicated that knowledge gaps regarding lifecycle impact should not be retained, as they did not perceive it as an immediate or clearly recognized barrier in current practice compared with more direct concerns such as cost, availability, and client preferences.

**Table 4:** Retention of barriers and strategies.

Code	Item	Retain (count)		Retain (%)	
		Yes	No	Yes	No
B1	Aesthetic limitations	21	1	95	5
B2	Complex supply chains	19	3	86	14
B3	Cultural resistance	20	2	91	9
B4	Greenwashing issues	16	6	73	27
B5	Hidden health risks	18	4	82	18
B6	Higher initial costs	19	3	86	14
B7	Inadequate testing and certification	20	2	91	9
B8	Inconsistent standards	18	4	82	18
B9	Insufficient research and development	18	4	82	18
B10	Knowledge gaps regarding lifecycle impact	14	8	64	36
B11	Lack of awareness	21	1	95	5
B12	Lack of collaboration	21	1	95	5
B13	Limited availability of sources	19	3	86	14
B14	Limited client interest	22	0	100	0
B15	Limited data availability	18	4	82	18
B16	Performance concerns	18	4	82	18
B17	Regulatory hurdles	21	1	95	5
B18	Shortage of skills	20	2	91	9
B19	Time constraints	18	4	82	18
S1	Conduct life cycle cost analysis	22	0	100	0
S2	Develop client education initiatives	22	0	100	0
S3	Develop comprehensive resource databases	21	1	95	5
S4	Encourage cultural shift	21	1	95	5
S5	Establish a supplier network	22	0	100	0
S6	Establish IAQ monitoring protocols	20	2	91	9
S7	Foster a collaborative design culture	22	0	100	0
S8	Foster interdisciplinary collaboration	20	2	91	9
S9	Implement certification mandates	21	1	95	5
S10	Implement reuse and upcycling programs	22	0	100	0
S11	Invest in educational campaigns	21	1	95	5
S12	Invest in R&D initiatives	22	0	100	0
S13	Promote third-party testing and certification	22	0	100	0
S14	Provide streamlined decision-making processes	21	1	95	5
S15	Streamline procurement processes	22	0	100	0

#### 4.1.2 Round 2

Following the retention stage, the Delphi process moved to the second round, which focused on assessing the relative importance of the identified barriers and strategies. Of the 22 experts who participated in Round 1, 16 experts continued to Round 2. The reduction in panel size was due to natural attrition: the 6 experts who did not participate in Round 2 failed to complete the questionnaire within the designated response period and were not excluded by the research team based on any eligibility criterion. A dropout rate of approximately 27% (6 out of 22) is common and considered acceptable in multi-round Delphi studies, which typically tolerate up to 30% attrition between rounds (40). The 16 remaining experts continued to represent a diverse and highly qualified cross-section of the industry, and this final panel size remains well within the recommended range of 10 to 18 experts for Delphi-based decision-making studies (38, 39). While the possibility of non-response bias cannot be entirely ruled out, the professional and academic diversity of the final panel, as documented in Table 3, provides reasonable assurance that attrition did not systematically skew the findings toward any particular professional perspective. In this round, the questionnaire was circulated to the remaining panelists, who were asked to rate the extent to which each barrier and strategy influences the adoption of non-toxic materials in interior design. The responses were then compiled and statistically analyzed to determine the relative weight of each factor. To evaluate the level of agreement among the experts, SD was used to measure response dispersion. As shown in Table 5, the SD values for all items remained below the accepted threshold of 1.64, indicating an acceptable level of consensus across the panel. This confirms that the experts’ judgments were sufficiently consistent to support interpretation of the mean scores. In this context, the mean values represent the perceived significance of each barrier and strategy, with higher scores indicating stronger influence on the adoption of non-toxic materials. Among the identified barriers, B14: limited client interest received the highest mean score (Mean = 4.13), making it the most influential barrier according to the expert panel. This was followed by B2: complex supply chains (Mean = 4.00). A second group of barriers also received comparatively high ratings, including B3: Cultural Resistance, B5: hidden health risks, B8: inconsistent standards, B13: limited availability of sources, and B17: regulatory hurdles, each with a mean score of 3.88. These findings suggest that the adoption of non-toxic materials is strongly influenced by a combination of client- and market-related factors and regulatory challenges. Other barriers, such as B1: aesthetic limitations and B18: shortage of skills, were also considered important, both recording a mean of 3.81, while B11: lack of awareness achieved a mean of 3.75. By contrast, B4: greenwashing issues received the lowest mean score among all barriers (Mean = 2.88), indicating that although it was retained in the previous round, it was regarded as less influential than the other barriers. Similarly, B19: time constraints recorded a relatively low score (Mean = 3.25), suggesting it was perceived as a less critical issue compared with factors such as client interest, supply chains, and regulatory concerns.

**Table 5:** Second round of Delphi survey results.

Code	Item	Mean	SD	Median
B1	Aesthetic limitations	3.81	1.33	4.5
B2	Complex supply chains	4.00	1.06	4
B3	Cultural resistance	3.88	1.22	4.5
B4	Greenwashing issues	2.88	1.11	3
B5	Hidden health risks	3.88	1.36	4
B6	Higher initial costs	3.69	1.21	4
B7	Inadequate testing and certification	3.50	1.17	3
B8	Inconsistent standards	3.88	0.99	4
B9	Insufficient research and development	3.69	0.92	3.5
B10	Knowledge gaps regarding lifecycle impact	3.69	1.31	4
B11	Lack of awareness	3.75	0.97	4
B12	Lack of collaboration	3.63	1.17	3.5
B13	Limited availability of sources	3.88	1.05	4
B14	Limited client interest	4.13	1.05	4.5
B15	Limited data availability	3.56	1.12	3
B16	Performance concerns	3.69	1.21	4
B17	Regulatory hurdles	3.88	1.05	4
B18	Shortage of skills	3.81	1.24	4
B19	Time constraints	3.25	1.35	3
S1	Conduct life cycle cost analysis	3.81	1.33	4
S2	Develop client education initiatives	4.06	1.20	4.5
S3	Develop comprehensive resource databases	4.13	1.17	4
S4	Encourage cultural shift	3.88	1.17	4
S5	Establish a supplier network	3.74	1.17	4
S6	Establish IAQ monitoring protocols	3.72	1.37	3.5
S7	Foster a collaborative design culture	3.88	1.41	4.5
S8	Foster interdisciplinary collaboration	4.00	1.27	4.5
S9	Implement certification mandates	3.81	1.18	4
S10	Implement reuse and upcycling programs	3.71	1.22	3.5
S11	Invest in educational campaigns	4.00	1.32	4.5
S12	Invest in R&D initiatives	4.06	1.20	4.5
S13	Promote third-party testing and certification	3.94	1.30	4.5
S14	Provide streamlined decision-making processes	3.86	1.30	4
S15	Streamline procurement processes	3.81	1.38	4

With regard to the strategies, S3: develop comprehensive resource databases emerged as the highest-rated strategy (Mean = 4.13), indicating that access to reliable, consolidated information was viewed as a particularly important measure to support the adoption of non-toxic materials. This was followed closely by S2: develop client education initiatives and S12: invest in R&D initiatives, both of which recorded a mean of 4.06 and a median of 4.5. These results highlight the strong importance placed by the experts on information provision, client engagement, and innovation-driven support mechanisms. Other highly rated strategies included S8: foster interdisciplinary collaboration and S11: invest in educational campaigns, each with a mean of 4.00, as well as S13: promote third-party testing and certification (Mean = 3.94). In addition, S4: encourage cultural shift and S7: foster a collaborative design culture both recorded a mean of 3.88, further emphasizing the perceived value of collaborative and mindset-oriented interventions. Although all strategies were rated positively overall, S6: establish IAQ monitoring protocols (Mean = 3.72) and S10: implement reuse and upcycling programs (Mean = 3.71) were among the comparatively lower-rated strategies.

### 4.2 Ranking of barriers and strategies

After completing the Delphi survey, a decision matrix was developed using the scores assigned by the expert panel. In this matrix, each row represented an individual barrier or strategy, while each column corresponded to the experts’ evaluations. Organizing the data in this way offered a clear and systematic overview of the panel’s judgments, making it easier to observe patterns across the items and providing the basis for subsequent analysis. The matrix was then transformed into a normalized decision matrix using Equation 1. The normalized results are presented in Supplementary Table S1 for barriers and Supplementary Table S4 for strategies. To construct the weighted-normalized decision matrix, it was first necessary to determine the relative importance of each expert’s opinion. Accordingly, expert weights were calculated based on an approach adopted from previous studies (48, 49). These weights were derived from the point-based expert qualification system established during the Delphi process. Specifically, the weight assigned to each expert was calculated as the expert’s total points divided by the sum of points awarded to all experts. For example, if Expert A received 14 points and the total points across all experts amounted to 315, the resulting weight would be 14/315 = 0.04. The calculated expert weights are presented in Table 3. These weights were subsequently applied using Equation 2 to produce the weighted-normalized decision matrix, which is reported in Supplementary Table S2 for barriers and Supplementary Table S5 for strategies.

Next, the positive and negative ideal solutions were identified using Equations 3, 4, respectively. These results are provided in Supplementary Table S3 for barriers and Supplementary Table S6 for strategies. The positive ideal solution represents the most desirable condition, defined by the maximum weighted normalized value for beneficial criteria and the minimum for non-beneficial criteria. Conversely, the negative ideal solution represents the least desirable condition, characterized by the minimum weighted normalized value for beneficial criteria and the maximum for non-beneficial criteria. Using Equations 5–7, the separation measures from the positive ideal solution $\left(D_{i}^{+}\right)$ , the negative ideal solution $\left(D_{i}^{-}\right)$ , and the relative closeness to the ideal solution $\left(C_{i}\right)$ were computed for each factor. The resulting values are presented in Table 6. The $C_{i}$ values range from 0 to 1 and reflect the extent to which each factor is positioned closer to the ideal solution. A higher $C_{i}$ value indicates greater proximity to the ideal condition and, consequently, a higher priority in the final ranking.

**Table 6:** Ranking of barriers and strategies.

Code	Item	Di+	Di-	Ci	Rank
Barriers					
B11	Lack of awareness	0.000005	0.000008	0.616	1
B14	Limited client interest	0.000006	0.000008	0.589	2
B3	Cultural resistance	0.000006	0.000007	0.533	3
B12	Lack of collaboration	0.000005	0.000005	0.531	4
B15	Limited data availability	0.000007	0.000006	0.465	5
B7	Inadequate testing and certification	0.000009	0.000006	0.424	6
B8	Inconsistent standards	0.000008	0.000004	0.358	7
B9	Insufficient research and development	0.000008	0.000004	0.342	8
B18	Shortage of skills	0.000008	0.000004	0.341	9
B6	Higher initial costs	0.000010	0.000004	0.272	10
B1	Aesthetic limitations	0.000010	0.000003	0.248	11
B10	Knowledge gaps regarding lifecycle impact	0.000010	0.000003	0.236	12
B13	Limited availability of sources	0.000009	0.000003	0.233	13
B17	Regulatory hurdles	0.000010	0.000003	0.232	14
B16	Performance concerns	0.000011	0.000003	0.202	15
B5	Hidden health risks	0.000015	0.000002	0.115	16
B4	Greenwashing issues	0.000013	0.000002	0.145	17
B19	Time constraints	0.000013	0.000002	0.113	18
B2	Complex supply chains	0.001062	0.000014	0.013	19
Strategies					
S11	Invest in educational campaigns	0.000001	0.000011	0.924	1
S2	Develop client education initiatives	0.000001	0.000011	0.882	2
S7	Foster a collaborative design culture	0.000002	0.000010	0.867	3
S8	Foster interdisciplinary collaboration	0.000002	0.000010	0.841	4
S3	Develop comprehensive resource databases	0.000002	0.000010	0.835	5
S4	Encourage cultural shift	0.000002	0.000010	0.828	6
S12	Invest in R&D initiatives	0.000005	0.000008	0.609	7
S9	Implement certification mandates	0.000006	0.000007	0.532	8
S14	Provide streamlined decision-making processes	0.000007	0.000006	0.446	9
S13	Promote third-party testing and certification	0.000007	0.000005	0.440	10
S5	Establish a supplier network	0.000007	0.000005	0.415	11
S15	Streamline procurement processes	0.000008	0.000005	0.371	12
S6	Establish IAQ monitoring protocols	0.000009	0.000005	0.367	13
S1	Conduct life cycle cost analysis	0.000010	0.000001	0.052	14
S10	Implement reuse and upcycling programs	0.000851	0.000004	0.004	15

**Figure 2:**
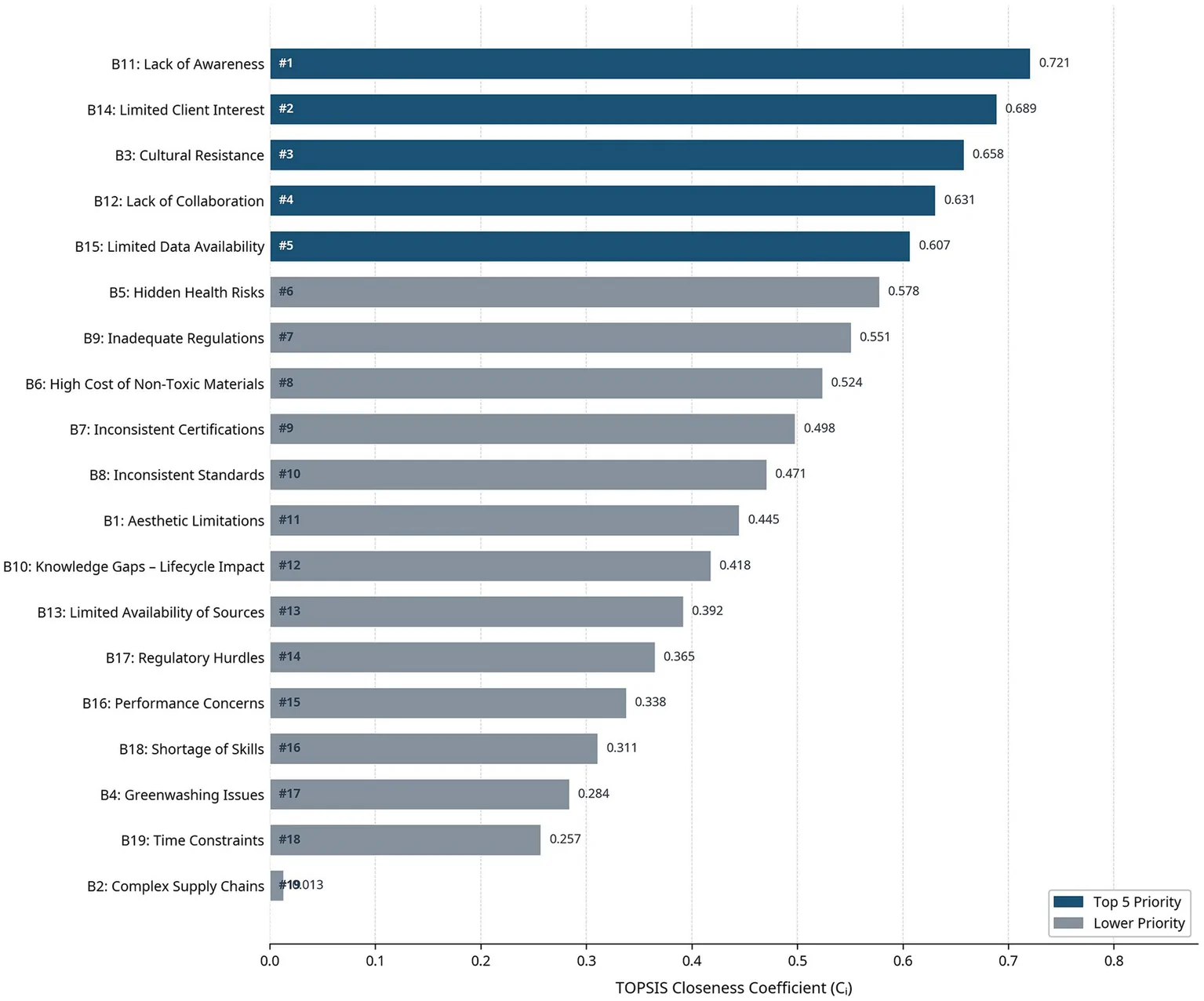
Priority ranking of barriers.

**Figure 3:**
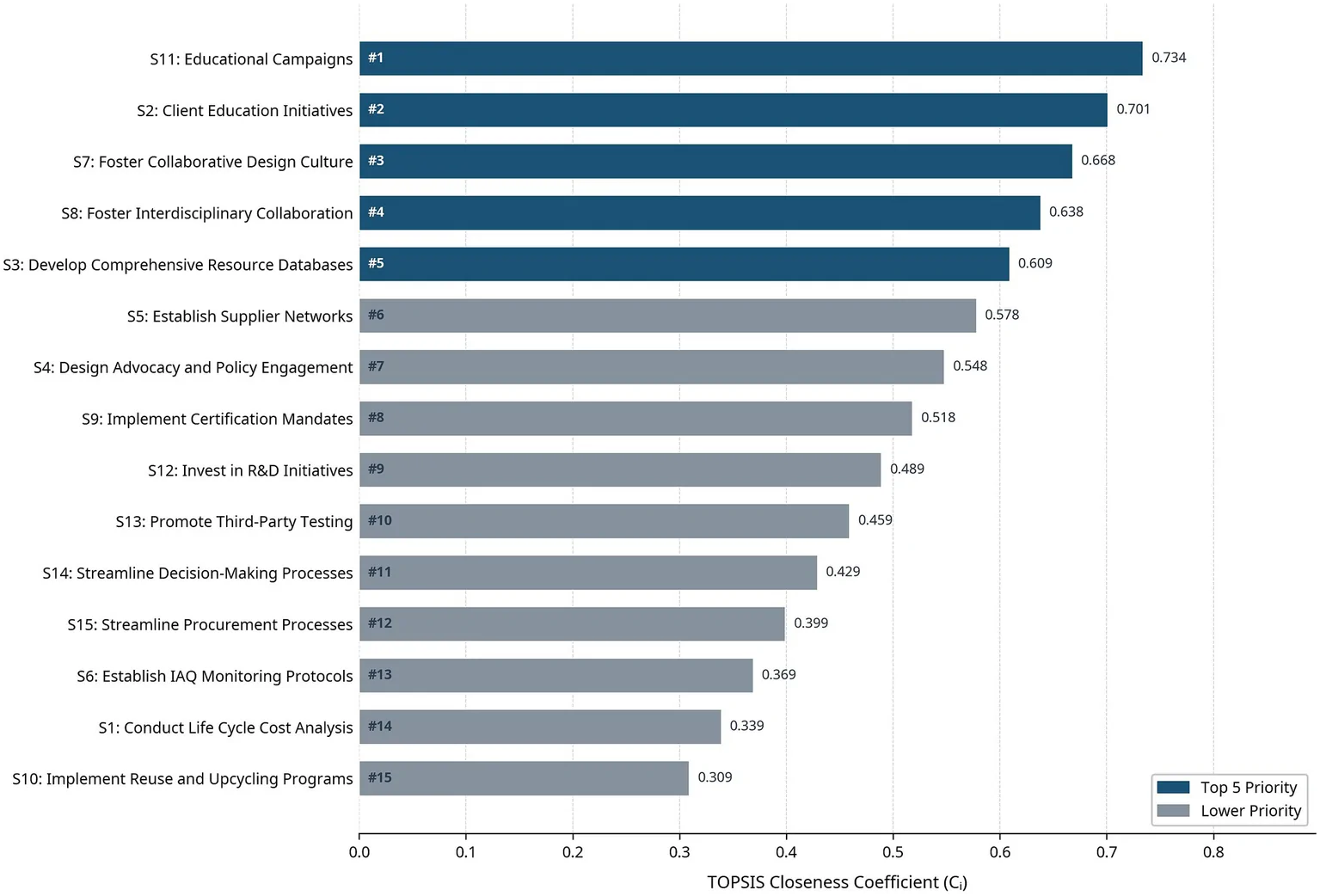
Priority ranking of strategies.

Table 6 presents the final ranking of the identified barriers and strategies based on the TOPSIS analysis. The ranking indicates the relative priority of each item, with those placed higher in the list considered more influential for barriers and more important for strategies to support the adoption of non-toxic materials in interior design. For the barriers (see Figure 2), B11: lack of awareness emerged as the highest-ranked barrier, followed by B14: limited client interest, B3: cultural resistance, B12: lack of collaboration, and B15: limited data availability. The lower-ranked barriers included B1: Aesthetic Limitations (rank 11), B10: knowledge gaps regarding lifecycle impact (rank 12), B13: limited availability of sources (rank 13), and B17: regulatory hurdles (rank 14). Meanwhile, B16: performance concerns were ranked 15th, followed by B4: greenwashing issues (rank 17) and B19: time constraints (rank 18). The lowest-ranked barrier was B2: complex supply chains, which was placed 19th. For the strategies (see Figure 3), S11: invest in educational campaigns was ranked first, followed by S2: develop client education initiatives, S7: Foster a collaborative design culture, S8: foster interdisciplinary collaboration, and S3: develop comprehensive resource databases. The lower-ranked strategies were S15: streamline procurement processes (rank 12), S6: establish IAQ monitoring protocols (rank 13), and S1: conduct life cycle cost analysis (rank 14). The lowest-ranked strategy, S10: implement reuse and upcycling programs, ranked 15th.

## 5 Discussion

The present study examined the barriers to and strategies for adopting non-toxic materials in interior design through a combination of a comprehensive literature review, Delphi validation, and TOPSIS prioritization. One of the most significant findings of the study is that lack of awareness was identified as the highest-ranked barrier. This result suggests that limited knowledge among designers, clients, and other stakeholders remains the most fundamental obstacle to the adoption of non-toxic materials. In practice, awareness influences whether stakeholders recognize the health and environmental implications of material choices, understand the benefits of safer alternatives, and feel confident in specifying or approving such materials. When awareness is limited, non-toxic materials may be overlooked in favor of familiar conventional products, even when safer options are available. This finding is consistent with earlier research, which has shown that insufficient understanding of sustainable and healthy materials often hinders their wider application in design and construction practice (25, 26). Previous studies have similarly emphasized that material selection in the sustainable built environment is often constrained by limited professional and client knowledge, particularly where the health impacts of interior finishes and furnishings are not fully appreciated (26). In this sense, a lack of awareness can be seen as a root barrier that reinforces several other barriers identified in the study.

The high ranking of limited client interest further highlights the importance of demand-side factors in shaping material adoption decisions. Although designers may be willing to specify non-toxic materials, their ability to do so is often influenced by client preferences, priorities, and budget considerations. In many projects, clients place greater emphasis on immediate cost, aesthetics, and project timelines than on long-term health and environmental outcomes. As a result, non-toxic materials may be perceived as optional rather than essential. This finding is consistent with the literature, which has repeatedly identified client reluctance and weak market demand as major barriers to the adoption of sustainable materials in the built environment (26, 27). The result also helps explain why client-focused strategies, such as developing client education initiatives, were ranked among the most important responses. If clients do not understand the value of non-toxic materials, they are unlikely to support their specification, regardless of the designer’s intentions. Another key finding is the prominence of cultural resistance. This suggests that the challenge of adopting non-toxic materials is not only informational but also cultural. Established design practices, long-standing preferences for conventional materials, and resistance to changing familiar workflows can all discourage experimentation with healthier alternatives. Designers and project teams may prefer materials with which they are already familiar, especially when they are perceived to have predictable performance, visual appeal, and market acceptance. This tendency is well documented in previous studies, which have noted that transitions toward sustainable and health-oriented design practices are often slowed by habit, traditional mindsets, and reluctance to depart from conventional approaches (25, 28). The present finding, therefore, indicates that promoting non-toxic materials requires more than technical guidance; it also requires a gradual shift in professional values, perceptions, and norms.

The ranking of lack of collaboration as the fourth most important barrier also deserves attention. Material selection in interior design is rarely the responsibility of a single individual. Instead, it is shaped by interactions among designers, clients, architects, contractors, consultants, and suppliers. Where collaboration is weak, opportunities to discuss healthier alternatives may be missed, information may not be shared effectively, and procurement decisions may not align with design intentions. In the absence of such collaboration, even well-informed designers may face difficulties in implementing non-toxic materials due to fragmented communication and misaligned priorities across the project team (25, 27). Similarly, limited data availability emerged as a highly ranked barrier, underscoring the importance of information transparency and accessibility. Designers require clear and reliable data on material composition, health impacts, certifications, performance, and sourcing to make informed decisions (50). When such information is incomplete, inconsistent, or difficult to access, practitioners may be less willing to select non-toxic alternatives. It also reflects broader concerns in the built environment literature regarding the transparency of product information and the difficulty of comparing materials based on health-related criteria (31). An important point arising from the findings is that some barriers commonly assumed to be critical were ranked lower than expected. For example, complex supply chains received one of the highest mean scores in the Delphi round but were ranked last in the TOPSIS results. This significant divergence between the two methods is a methodologically important finding that warrants explicit explanation. The shift in ranking reflects the fundamental difference between the independent, absolute evaluation logic of the Delphi method and the relative, multi-attribute evaluation logic of TOPSIS. In the Delphi round, experts assess each barrier independently on an absolute scale, and the high mean score for B2 reflects broad and consistent recognition among practitioners that supply chain complexity is a real, operationally visible challenge. TOPSIS, however, evaluates all barriers simultaneously by computing each item’s geometric distance from the positive and negative ideal solutions, while applying the differential qualification weights assigned to each expert. Two interrelated mechanisms explain B2’s low TOPSIS rank. First, the application of expert weights may have shifted the relative influence of different rater profiles. Experts with higher qualification weights may have assigned lower scores to B2 relative to more fundamental, root-cause barriers such as Lack of Awareness (B11). In contrast, practitioners with direct project experience may have rated supply chain issues highly because they are immediately visible and operationally disruptive. When qualification weights are applied, the former group’s perspective carries greater mathematical influence, which can substantially alter the final ranking. Second, and more substantively, the TOPSIS result suggests that complex supply chains may function more as a downstream consequence of broader systemic weaknesses than as an independent root-cause barrier. When client demand is low, awareness is limited, and information systems are inadequate, the market for non-toxic materials remains underdeveloped, thereby constraining supply chain diversity and availability. In this sense, B2 is partly a symptom of more fundamental barriers rather than a primary driver of non-adoption. TOPSIS, by evaluating all barriers simultaneously within a weighted multi-attribute framework, effectively captures this systemic interdependency in a way that the independent Delphi evaluation cannot. This finding, therefore, does not contradict the Delphi results; rather, it enriches the interpretation by revealing that while supply chain complexity is a widely recognized practical obstacle, it may not be the most strategically central barrier for intervention when considered in relation to the full set of systemic, cultural, and informational challenges. Likewise, barriers such as greenwashing issues and time constraints were ranked relatively low, indicating that while they are relevant, they may be perceived as secondary compared with more structural barriers such as awareness, collaboration, and client interest. This does not diminish their importance; rather, it suggests that the experts viewed them as less central to the adoption problem.

The strategy rankings further reinforce the interpretation that educational and collaborative responses are the most effective ways to address the identified barriers. The highest-ranked strategy, investing in educational campaigns, directly addresses the problem of limited awareness. Educational campaigns can improve understanding among both professionals and clients regarding the health risks associated with conventional materials and the benefits of safer alternatives. They can also help dispel misconceptions related to cost, performance, and aesthetics. This result is strongly supported by previous studies, which have argued that improving awareness through workshops, training programs, and broader communication efforts is essential for increasing the uptake of sustainable and healthy materials (25, 26). The second-ranked strategy, develop client education initiatives, is closely aligned with the high ranking of limited client interest. This indicates that the experts viewed client engagement as a priority area for intervention. Client education initiatives can equip decision-makers with a clearer understanding of how non-toxic materials contribute to healthier indoor environments, user wellbeing, and long-term value. Such initiatives may include project-specific presentations, evidence-based comparisons, design demonstrations, and communication materials tailored to client needs and concerns (25, 27). In this respect, the study highlights the central role of designers not only as specifiers but also as communicators and advocates for healthier material choices.

The high ranking of fostering a collaborative design culture and interdisciplinary collaboration further supports the argument that adopting non-toxic materials depends on collective action among multiple stakeholders. These strategies are particularly relevant to addressing the barrier of lack of collaboration, but they also have broader value in improving information exchange, aligning project goals, and supporting innovation. A collaborative design culture can encourage material health considerations to be discussed earlier in the project lifecycle, while interdisciplinary collaboration can bring together expertise from design, construction, procurement, environmental assessment, and material science (51). Prior literature has similarly emphasized that sustainable and health-oriented design outcomes are more likely when project participants work together from the early stages of design and decision-making (25, 27). Another notable finding is the strong position of developing comprehensive resource databases. This strategy directly addresses the barrier of limited data availability by making information on non-toxic materials more accessible, structured, and usable in practice. Comprehensive databases can support designers by providing consolidated information on material ingredients, certifications, performance characteristics, lifecycle implications, and supplier options. Such tools reduce uncertainty and can help practitioners compare alternatives more efficiently. This finding aligns with previous research identifying the need for practical decision-support systems and improved access to material information to facilitate sustainable design choices (52).

The relatively lower ranking of strategies such as conducting life cycle cost analysis and implementing reuse and upcycling programs is also noteworthy. Although these strategies remain relevant, the results indicate that they were not perceived as the most immediate or influential responses to the barriers identified in this study. Life cycle cost analysis can help demonstrate long-term economic benefits, but it may be less effective where the primary obstacles are a lack of awareness and weak client demand rather than purely financial concerns. Similarly, reuse and upcycling are valuable from a sustainability perspective, but they may not directly address the specific challenge of reducing toxicity in interior materials unless accompanied by careful evaluations of health and performance. This distinction is important because it shows that not all sustainability-oriented strategies are equally effective at promoting the adoption of non-toxic materials. Instead, strategies must be closely aligned with the specific barriers present in the decision context.

## 6 Implications

This study offers implications more specifically tied to the Indian interior design context and the priorities identified through the Delphi–TOPSIS analysis. Theoretically, it contributes to the literature by shifting the discussion on non-toxic materials from the broad built environment to the more specific domain of interior design, where direct and prolonged human exposure to finishes, furnishings, adhesives, coatings, and engineered products makes material health especially important. By focusing on India, the study also adds contextual value to a field that has largely been discussed in generalized or international terms, with limited attention to the realities of developing economies. The study demonstrates that barriers to the adoption of non-toxic materials are shaped not only by technical limitations but also by market conditions, client attitudes, professional habits, and weak information systems. This helps reposition the issue as a context-dependent adoption challenge rather than merely a material selection problem. In practical terms, the findings have direct implications for key stakeholders in India’s interior design and built environment sectors. For interior designers and design firms, the results indicate that improving adoption will require more than individual willingness alone; it will require greater knowledge of specifications, better access to verified product information, and more effective communication with clients about the health benefits of non-toxic materials. Since client awareness and demand were identified as critical concerns, the findings suggest that practitioners must play a more active role in educating clients and translating health benefits into understandable design and lifecycle outcomes. For suppliers and manufacturers, the study highlights the need for clearer product disclosure, credible certification, and transparent communication regarding material contents and emissions, especially in a market where misleading environmental claims may create confusion. For design educators and training institutions in India, the study points to the need to incorporate material toxicity, indoor environmental health, and product assessment skills into interior design education so that future professionals are better prepared to make health-informed material decisions. For policymakers, standards bodies, and professional associations, the findings indicate that adoption can be strengthened through context-specific guidelines, healthier material databases, improved labeling and disclosure practices, and institutional support mechanisms that reduce the burden on individual practitioners. In this way, the study provides an evidence-based foundation for targeted interventions that are more responsive to the realities of interior design practice in India and other developing-region contexts. Beyond India, these findings are also relevant to regions where the adoption of healthier interior materials is constrained by similar structural challenges, even if the regulatory and market conditions differ. In such contexts, the prioritized strategies identified in this study may serve as adaptable guidance for improving indoor environmental quality through better material selection. However, their implementation should be tailored to local market maturity, professional practices, and policy environments. In this way, the study provides an evidence-based foundation for targeted interventions that are responsive not only to the realities of interior design practice in India but also to comparable settings internationally.

## 7 Conclusion

This study identified and prioritized the barriers to and strategies for adopting non-toxic materials in interior design through a Comprehensive Literature Review, Delphi expert validation, and TOPSIS multi-attribute decision-making analysis. The findings demonstrate that the challenge of transitioning to non-toxic materials is fundamentally informational, cultural, and organizational in nature, rather than purely technical or economic. More specifically, the findings indicate that adoption is often hindered by limited understanding of material health issues, weak client demand for healthier alternatives, resistance to changing familiar professional practices, fragmented stakeholder coordination, and insufficient access to reliable information on materials. The five most critical barriers to non-toxic material adoption, in order of priority, are:

- Lack of awareness
- Limited client interest
- Cultural resistance
- Lack of collaboration
- Limited data availability

These barriers indicate that the adoption problem is not simply about whether non-toxic materials exist, but whether designers, clients, suppliers, and other stakeholders have the knowledge, motivation, and support systems needed to specify and implement them effectively. The five highest-priority strategies for overcoming these barriers, in order of importance, are:

- Educational campaigns
- Client education initiatives
- Fostering a collaborative design culture
- Interdisciplinary collaboration
- Developing comprehensive resource databases

These strategies directly address the identified barriers by strengthening awareness, improving client understanding, encouraging shared decision-making, and making trustworthy information easier to access and apply in practice. Together, they suggest that successful adoption depends on combining knowledge-building efforts with stronger communication and coordination across the project team. Investing in client education and cross-disciplinary collaboration from the outset of a project is the most effective and immediately actionable step practitioners can take to advance the adoption of non-toxic materials in interior design. This study contributes a structured, expert-validated framework for understanding and addressing the adoption challenge in the Indian context. At the same time, the findings have broader relevance to other developing or transitional contexts where similar barriers to the adoption of healthier materials may exist. Future research should test the implementation of these prioritized strategies across specific project types, compare perspectives among stakeholder groups, and examine the framework’s transferability to other developing-economy contexts.

Despite its contributions, this study has several limitations that should be acknowledged. First, the study relied on expert judgment through the Delphi technique and TOPSIS analysis, so the findings are influenced by the participants’ experience, knowledge, and perspectives. Although expert-based methods are valuable for prioritization, results may vary across expert groups or when larger samples are used. Second, the final Round 2 analysis was based on 16 of the 22 qualified Round 1 experts due to panel attrition. Although this final panel size remains acceptable for Delphi and MADM applications, the reduction may have introduced some degree of non-response bias if the opinions of the six non-completing experts differed systematically from those who remained. Third, the study focused on identifying and ranking barriers and strategies rather than testing them through real-world implementation. As a result, the practical effectiveness of the proposed strategies was not empirically evaluated within actual interior design spaces. Fourth, the study addressed non-toxic materials in a general interior design context and did not extensively differentiate among project types, such as residential, commercial, healthcare, or educational interiors, where barriers and priorities may differ. Fifth, because this study is situated in the Indian context, its findings may reflect country-specific market conditions, professional practices, regulatory environments, and levels of awareness, which may limit the generalizability of the results beyond India or similar developing-region contexts. Future research could address these limitations by applying the proposed framework in specific project settings, comparing perspectives across different stakeholder groups, or testing the implementation of prioritized strategies in practice. Additional studies may also explore regional differences, policy influences, and the role of certification systems in greater depth. Such efforts would further strengthen the understanding of how non-toxic materials can be more effectively integrated into interior design practice.

## Data Availability

The raw data supporting the conclusions of this article will be made available by the authors, without undue reservation.
