# Supplementary material for: Prioritizing barriers and strategies for non-toxic material adoption in interior design
Source: Front Public Health. 2026 Sep 4;14:1901022. doi: 10.3389/fpubh.2026.1901022 (PMC13585527; doi:10.3389/fpubh.2026.1901022)
Supplement: Supplementary file 1 [file Table_1.DOCX]

Table S1. Normalized matrix for barriers.

| B1 | 0.0113 | 0.0127 | 0.0111 | 0.0141 | 0.0204 | 0.0134 | 0.0095 | 0.0162 | 0.0044 | 0.0145 | 0.0254 | 0.0194 | 0.0122 | 0.0129 | 0.0198 | 0.0116 |
| --- | --- | --- | --- | --- | --- | --- | --- | --- | --- | --- | --- | --- | --- | --- | --- | --- |
| B1 | 0.0156 | 0.0177 | 0.0121 | 0.0238 | 0.0500 | 0.0326 | 0.0218 | 0.0205 | 0.0263 | 0.0147 | 0.0211 | 0.0200 | 0.0104 | 0.0183 | 0.0222 | 0.0153 |
| B3 | 0.0068 | 0.0127 | 0.0111 | 0.0176 | 0.0204 | 0.0268 | 0.0159 | 0.0162 | 0.0219 | 0.0058 | 0.0254 | 0.0129 | 0.0122 | 0.0129 | 0.0119 | 0.0116 |
| B4 | 0.0091 | 0.0025 | 0.0111 | 0.0141 | 0.0204 | 0.0067 | 0.0127 | 0.0065 | 0.0088 | 0.0087 | 0.0254 | 0.0129 | 0.0073 | 0.0129 | 0.0119 | 0.0093 |
| B5 | 0.0050 | 0.0052 | 0.0049 | 0.0099 | 0.0189 | 0.0068 | 0.0092 | 0.0100 | 0.0220 | 0.0054 | 0.0085 | 0.0169 | 0.0051 | 0.0144 | 0.0108 | 0.0063 |
| B6 | 0.0113 | 0.0127 | 0.0111 | 0.0106 | 0.0204 | 0.0201 | 0.0159 | 0.0130 | 0.0044 | 0.0116 | 0.0169 | 0.0258 | 0.0122 | 0.0172 | 0.0119 | 0.0093 |
| B7 | 0.0113 | 0.0102 | 0.0111 | 0.0106 | 0.0204 | 0.0336 | 0.0095 | 0.0065 | 0.0132 | 0.0058 | 0.0169 | 0.0194 | 0.0122 | 0.0129 | 0.0159 | 0.0116 |
| B8 | 0.0113 | 0.0127 | 0.0111 | 0.0176 | 0.0204 | 0.0201 | 0.0127 | 0.0130 | 0.0132 | 0.0116 | 0.0169 | 0.0258 | 0.0122 | 0.0172 | 0.0119 | 0.0093 |
| B9 | 0.0113 | 0.0076 | 0.0111 | 0.0141 | 0.0204 | 0.0201 | 0.0127 | 0.0162 | 0.0132 | 0.0116 | 0.0254 | 0.0194 | 0.0122 | 0.0129 | 0.0119 | 0.0093 |
| B10 | 0.0113 | 0.0076 | 0.0111 | 0.0141 | 0.0204 | 0.0134 | 0.0159 | 0.0162 | 0.0088 | 0.0145 | 0.0169 | 0.0194 | 0.0122 | 0.0086 | 0.0159 | 0.0116 |
| B11 | 0.0156 | 0.0177 | 0.0152 | 0.0190 | 0.0375 | 0.0217 | 0.0175 | 0.0164 | 0.0158 | 0.0147 | 0.0211 | 0.0300 | 0.0104 | 0.0244 | 0.0222 | 0.0153 |
| B12 | 0.0113 | 0.0127 | 0.0067 | 0.0106 | 0.0306 | 0.0268 | 0.0032 | 0.0097 | 0.0175 | 0.0145 | 0.0254 | 0.0129 | 0.0122 | 0.0129 | 0.0159 | 0.0116 |
| B13 | 0.0156 | 0.0177 | 0.0152 | 0.0190 | 0.0250 | 0.0217 | 0.0131 | 0.0164 | 0.0263 | 0.0147 | 0.0316 | 0.0300 | 0.0138 | 0.0305 | 0.0167 | 0.0153 |
| B14 | 0.0113 | 0.0127 | 0.0111 | 0.0176 | 0.0204 | 0.0268 | 0.0127 | 0.0130 | 0.0088 | 0.0145 | 0.0254 | 0.0194 | 0.0122 | 0.0172 | 0.0198 | 0.0116 |
| B15 | 0.0113 | 0.0076 | 0.0111 | 0.0106 | 0.0204 | 0.0134 | 0.0127 | 0.0130 | 0.0088 | 0.0145 | 0.0254 | 0.0194 | 0.0122 | 0.0129 | 0.0119 | 0.0116 |
| B16 | 0.0113 | 0.0127 | 0.0111 | 0.0106 | 0.0204 | 0.0134 | 0.0127 | 0.0065 | 0.0175 | 0.0145 | 0.0254 | 0.0129 | 0.0122 | 0.0172 | 0.0119 | 0.0116 |
| B17 | 0.0113 | 0.0127 | 0.0111 | 0.0106 | 0.0204 | 0.0201 | 0.0159 | 0.0130 | 0.0219 | 0.0116 | 0.0169 | 0.0194 | 0.0098 | 0.0172 | 0.0119 | 0.0116 |
| B18 | 0.0113 | 0.0127 | 0.0111 | 0.0106 | 0.0204 | 0.0067 | 0.0159 | 0.0130 | 0.0175 | 0.0116 | 0.0169 | 0.0194 | 0.0122 | 0.0172 | 0.0159 | 0.0116 |
| B19 | 0.0063 | 0.0127 | 0.0049 | 0.0074 | 0.0189 | 0.0135 | 0.0069 | 0.0091 | 0.0044 | 0.0093 | 0.0142 | 0.0123 | 0.0083 | 0.0078 | 0.0095 | 0.0061 |

Table S2. Weighted-normalized matrix for barriers.

| B1 | 0.0005 | 0.0008 | 0.0008 | 0.0013 | 0.0010 | 0.0011 | 0.0005 | 0.0011 | 0.0002 | 0.0006 | 0.0010 | 0.0008 | 0.0012 | 0.0009 | 0.0014 | 0.0010 |
| --- | --- | --- | --- | --- | --- | --- | --- | --- | --- | --- | --- | --- | --- | --- | --- | --- |
| B1 | 0.0006 | 0.0011 | 0.0008 | 0.0021 | 0.0025 | 0.0026 | 0.0011 | 0.0014 | 0.0013 | 0.0006 | 0.0008 | 0.0008 | 0.0010 | 0.0013 | 0.0016 | 0.0014 |
| B3 | 0.0003 | 0.0008 | 0.0008 | 0.0016 | 0.0010 | 0.0021 | 0.0008 | 0.0011 | 0.0011 | 0.0002 | 0.0010 | 0.0005 | 0.0012 | 0.0009 | 0.0008 | 0.0010 |
| B4 | 0.0004 | 0.0002 | 0.0008 | 0.0013 | 0.0010 | 0.0005 | 0.0006 | 0.0005 | 0.0004 | 0.0003 | 0.0010 | 0.0005 | 0.0007 | 0.0009 | 0.0008 | 0.0008 |
| B5 | 0.0002 | 0.0003 | 0.0003 | 0.0009 | 0.0009 | 0.0005 | 0.0005 | 0.0007 | 0.0011 | 0.0002 | 0.0003 | 0.0007 | 0.0005 | 0.0010 | 0.0008 | 0.0006 |
| B6 | 0.0005 | 0.0008 | 0.0008 | 0.0010 | 0.0010 | 0.0016 | 0.0008 | 0.0009 | 0.0002 | 0.0005 | 0.0007 | 0.0010 | 0.0012 | 0.0012 | 0.0008 | 0.0008 |
| B7 | 0.0005 | 0.0006 | 0.0008 | 0.0010 | 0.0010 | 0.0027 | 0.0005 | 0.0005 | 0.0007 | 0.0002 | 0.0007 | 0.0008 | 0.0012 | 0.0009 | 0.0011 | 0.0010 |
| B8 | 0.0005 | 0.0008 | 0.0008 | 0.0016 | 0.0010 | 0.0016 | 0.0006 | 0.0009 | 0.0007 | 0.0005 | 0.0007 | 0.0010 | 0.0012 | 0.0012 | 0.0008 | 0.0008 |
| B9 | 0.0005 | 0.0005 | 0.0008 | 0.0013 | 0.0010 | 0.0016 | 0.0006 | 0.0011 | 0.0007 | 0.0005 | 0.0010 | 0.0008 | 0.0012 | 0.0009 | 0.0008 | 0.0008 |
| B10 | 0.0005 | 0.0005 | 0.0008 | 0.0013 | 0.0010 | 0.0011 | 0.0008 | 0.0011 | 0.0004 | 0.0006 | 0.0007 | 0.0008 | 0.0012 | 0.0006 | 0.0011 | 0.0010 |
| B11 | 0.0006 | 0.0011 | 0.0011 | 0.0017 | 0.0019 | 0.0017 | 0.0009 | 0.0011 | 0.0008 | 0.0006 | 0.0008 | 0.0012 | 0.0010 | 0.0017 | 0.0016 | 0.0014 |
| B12 | 0.0005 | 0.0008 | 0.0005 | 0.0010 | 0.0015 | 0.0021 | 0.0002 | 0.0007 | 0.0009 | 0.0006 | 0.0010 | 0.0005 | 0.0012 | 0.0009 | 0.0011 | 0.0010 |
| B13 | 0.0006 | 0.0011 | 0.0011 | 0.0017 | 0.0013 | 0.0017 | 0.0007 | 0.0011 | 0.0013 | 0.0006 | 0.0013 | 0.0012 | 0.0014 | 0.0021 | 0.0012 | 0.0014 |
| B14 | 0.0005 | 0.0008 | 0.0008 | 0.0016 | 0.0010 | 0.0021 | 0.0006 | 0.0009 | 0.0004 | 0.0006 | 0.0010 | 0.0008 | 0.0012 | 0.0012 | 0.0014 | 0.0010 |
| B15 | 0.0005 | 0.0005 | 0.0008 | 0.0010 | 0.0010 | 0.0011 | 0.0006 | 0.0009 | 0.0004 | 0.0006 | 0.0010 | 0.0008 | 0.0012 | 0.0009 | 0.0008 | 0.0010 |
| B16 | 0.0005 | 0.0008 | 0.0008 | 0.0010 | 0.0010 | 0.0011 | 0.0006 | 0.0005 | 0.0009 | 0.0006 | 0.0010 | 0.0005 | 0.0012 | 0.0012 | 0.0008 | 0.0010 |
| B17 | 0.0005 | 0.0008 | 0.0008 | 0.0010 | 0.0010 | 0.0016 | 0.0008 | 0.0009 | 0.0011 | 0.0005 | 0.0007 | 0.0008 | 0.0010 | 0.0012 | 0.0008 | 0.0010 |
| B18 | 0.0005 | 0.0008 | 0.0008 | 0.0010 | 0.0010 | 0.0005 | 0.0008 | 0.0009 | 0.0009 | 0.0005 | 0.0007 | 0.0008 | 0.0012 | 0.0012 | 0.0011 | 0.0010 |
| B19 | 0.0003 | 0.0008 | 0.0003 | 0.0007 | 0.0009 | 0.0011 | 0.0003 | 0.0006 | 0.0002 | 0.0004 | 0.0006 | 0.0005 | 0.0008 | 0.0005 | 0.0007 | 0.0006 |

Table S3. Ideal best and ideal worst for barriers.

| A+ | 0.0006 | 0.0011 | 0.0011 | 0.0021 | 0.0025 | 0.0027 | 0.0011 | 0.0014 | 0.0013 | 0.0006 | 0.0013 | 0.0012 | 0.0014 | 0.0021 | 0.0016 | 0.0014 |
| --- | --- | --- | --- | --- | --- | --- | --- | --- | --- | --- | --- | --- | --- | --- | --- | --- |
| A- | 0.0002 | 0.0002 | 0.0003 | 0.0007 | 0.0009 | 0.0005 | 0.0002 | 0.0005 | 0.0002 | 0.0002 | 0.0003 | 0.0005 | 0.0005 | 0.0005 | 0.0007 | 0.0006 |

Table S4. Normalized matrix for strategies.

| S1 | 0.0054 | 0.0054 | 0.0054 | 0.0091 | 0.0247 | 0.0286 | 0.0114 | 0.0060 | 0.0054 | 0.0054 | 0.0357 | 0.0137 | 0.0054 | 0.0100 | 0.0056 | 0.0074 |
| --- | --- | --- | --- | --- | --- | --- | --- | --- | --- | --- | --- | --- | --- | --- | --- | --- |
| S2 | 0.0141 | 0.0141 | 0.0141 | 0.0138 | 0.0411 | 0.0556 | 0.0216 | 0.0161 | 0.0145 | 0.0145 | 0.0351 | 0.0163 | 0.0141 | 0.0225 | 0.0161 | 0.0139 |
| S3 | 0.0054 | 0.0054 | 0.0054 | 0.0087 | 0.0247 | 0.0571 | 0.0088 | 0.0075 | 0.0054 | 0.0054 | 0.0308 | 0.0240 | 0.0054 | 0.0101 | 0.0056 | 0.0105 |
| S4 | 0.0141 | 0.0141 | 0.0141 | 0.0138 | 0.0274 | 0.0556 | 0.0172 | 0.0161 | 0.0145 | 0.0145 | 0.0351 | 0.0244 | 0.0141 | 0.0225 | 0.0129 | 0.0105 |
| S5 | 0.0141 | 0.0141 | 0.0141 | 0.0138 | 0.0274 | 0.0556 | 0.0172 | 0.0129 | 0.0145 | 0.0145 | 0.0351 | 0.0244 | 0.0141 | 0.0225 | 0.0161 | 0.0174 |
| S6 | 0.0141 | 0.0141 | 0.0141 | 0.0138 | 0.0274 | 0.0278 | 0.0216 | 0.0161 | 0.0145 | 0.0145 | 0.0351 | 0.0244 | 0.0141 | 0.0169 | 0.0161 | 0.0174 |
| S7 | 0.0141 | 0.0141 | 0.0141 | 0.0184 | 0.0274 | 0.0278 | 0.0129 | 0.0161 | 0.0145 | 0.0145 | 0.0175 | 0.0244 | 0.0141 | 0.0225 | 0.0161 | 0.0139 |
| S8 | 0.0141 | 0.0141 | 0.0141 | 0.0184 | 0.0274 | 0.0556 | 0.0129 | 0.0161 | 0.0145 | 0.0145 | 0.0351 | 0.0163 | 0.0141 | 0.0225 | 0.0161 | 0.0174 |
| S9 | 0.0141 | 0.0141 | 0.0141 | 0.0138 | 0.0411 | 0.0556 | 0.0086 | 0.0129 | 0.0145 | 0.0145 | 0.0351 | 0.0244 | 0.0141 | 0.0169 | 0.0129 | 0.0174 |
| S10 | 0.0141 | 0.0141 | 0.0141 | 0.0184 | 0.0274 | 0.0278 | 0.0216 | 0.0161 | 0.0145 | 0.0145 | 0.0526 | 0.0244 | 0.0141 | 0.0169 | 0.0129 | 0.0139 |
| S11 | 0.0141 | 0.0141 | 0.0141 | 0.0230 | 0.0274 | 0.0278 | 0.0129 | 0.0161 | 0.0145 | 0.0145 | 0.0351 | 0.0325 | 0.0141 | 0.0169 | 0.0129 | 0.0174 |
| S12 | 0.0141 | 0.0141 | 0.0141 | 0.0184 | 0.0411 | 0.0556 | 0.0216 | 0.0161 | 0.0145 | 0.0145 | 0.0351 | 0.0163 | 0.0141 | 0.0169 | 0.0161 | 0.0139 |
| S13 | 0.0141 | 0.0141 | 0.0141 | 0.0184 | 0.0274 | 0.0278 | 0.0216 | 0.0129 | 0.0145 | 0.0116 | 0.0351 | 0.0244 | 0.0141 | 0.0169 | 0.0161 | 0.0174 |
| S14 | 0.0141 | 0.0141 | 0.0141 | 0.0184 | 0.0274 | 0.0278 | 0.0172 | 0.0161 | 0.0145 | 0.0145 | 0.0351 | 0.0244 | 0.0141 | 0.0225 | 0.0161 | 0.0174 |
| S15 | 0.0141 | 0.0141 | 0.0141 | 0.0184 | 0.0274 | 0.0278 | 0.0172 | 0.0161 | 0.0116 | 0.0145 | 0.0175 | 0.0244 | 0.0141 | 0.0169 | 0.0129 | 0.0174 |

Table S5. Weighted-normalized matrix for strategies.

| S1 | 0.0002 | 0.0003 | 0.0004 | 0.0008 | 0.0012 | 0.0023 | 0.0006 | 0.0004 | 0.0003 | 0.0002 | 0.0014 | 0.0005 | 0.0005 | 0.0007 | 0.0004 | 0.0007 |
| --- | --- | --- | --- | --- | --- | --- | --- | --- | --- | --- | --- | --- | --- | --- | --- | --- |
| S2 | 0.0006 | 0.0008 | 0.0010 | 0.0012 | 0.0021 | 0.0044 | 0.0011 | 0.0011 | 0.0007 | 0.0006 | 0.0014 | 0.0007 | 0.0014 | 0.0016 | 0.0011 | 0.0013 |
| S3 | 0.0002 | 0.0003 | 0.0004 | 0.0008 | 0.0012 | 0.0046 | 0.0004 | 0.0005 | 0.0003 | 0.0002 | 0.0012 | 0.0010 | 0.0005 | 0.0007 | 0.0004 | 0.0009 |
| S4 | 0.0006 | 0.0008 | 0.0010 | 0.0012 | 0.0014 | 0.0044 | 0.0009 | 0.0011 | 0.0007 | 0.0006 | 0.0014 | 0.0010 | 0.0014 | 0.0016 | 0.0009 | 0.0009 |
| S5 | 0.0006 | 0.0008 | 0.0010 | 0.0012 | 0.0014 | 0.0044 | 0.0009 | 0.0009 | 0.0007 | 0.0006 | 0.0014 | 0.0010 | 0.0014 | 0.0016 | 0.0011 | 0.0016 |
| S6 | 0.0006 | 0.0008 | 0.0010 | 0.0012 | 0.0014 | 0.0022 | 0.0011 | 0.0011 | 0.0007 | 0.0006 | 0.0014 | 0.0010 | 0.0014 | 0.0012 | 0.0011 | 0.0016 |
| S7 | 0.0006 | 0.0008 | 0.0010 | 0.0017 | 0.0014 | 0.0022 | 0.0006 | 0.0011 | 0.0007 | 0.0006 | 0.0007 | 0.0010 | 0.0014 | 0.0016 | 0.0011 | 0.0013 |
| S8 | 0.0006 | 0.0008 | 0.0010 | 0.0017 | 0.0014 | 0.0044 | 0.0006 | 0.0011 | 0.0007 | 0.0006 | 0.0014 | 0.0007 | 0.0014 | 0.0016 | 0.0011 | 0.0016 |
| S9 | 0.0006 | 0.0008 | 0.0010 | 0.0012 | 0.0021 | 0.0044 | 0.0004 | 0.0009 | 0.0007 | 0.0006 | 0.0014 | 0.0010 | 0.0014 | 0.0012 | 0.0009 | 0.0016 |
| S10 | 0.0006 | 0.0008 | 0.0010 | 0.0017 | 0.0014 | 0.0022 | 0.0011 | 0.0011 | 0.0007 | 0.0006 | 0.0021 | 0.0010 | 0.0014 | 0.0012 | 0.0009 | 0.0013 |
| S11 | 0.0006 | 0.0008 | 0.0010 | 0.0021 | 0.0014 | 0.0022 | 0.0006 | 0.0011 | 0.0007 | 0.0006 | 0.0014 | 0.0013 | 0.0014 | 0.0012 | 0.0009 | 0.0016 |
| S12 | 0.0006 | 0.0008 | 0.0010 | 0.0017 | 0.0021 | 0.0044 | 0.0011 | 0.0011 | 0.0007 | 0.0006 | 0.0014 | 0.0007 | 0.0014 | 0.0012 | 0.0011 | 0.0013 |
| S13 | 0.0006 | 0.0008 | 0.0010 | 0.0017 | 0.0014 | 0.0022 | 0.0011 | 0.0009 | 0.0007 | 0.0005 | 0.0014 | 0.0010 | 0.0014 | 0.0012 | 0.0011 | 0.0016 |
| S14 | 0.0006 | 0.0008 | 0.0010 | 0.0017 | 0.0014 | 0.0022 | 0.0009 | 0.0011 | 0.0007 | 0.0006 | 0.0014 | 0.0010 | 0.0014 | 0.0016 | 0.0011 | 0.0016 |
| S15 | 0.0006 | 0.0008 | 0.0010 | 0.0017 | 0.0014 | 0.0022 | 0.0009 | 0.0011 | 0.0006 | 0.0006 | 0.0007 | 0.0010 | 0.0014 | 0.0012 | 0.0009 | 0.0016 |
|  |  |  |  |  |  |  |  |  |  |  |  |  |  |  |  |  |

Table S6. Ideal best and ideal worst for strategies.

| A+ | 0.0006 | 0.0008 | 0.0010 | 0.0021 | 0.0021 | 0.0046 | 0.0011 | 0.0011 | 0.0007 | 0.0006 | 0.0021 | 0.0013 | 0.0014 | 0.0016 | 0.0011 | 0.0016 |
| --- | --- | --- | --- | --- | --- | --- | --- | --- | --- | --- | --- | --- | --- | --- | --- | --- |
| A- | 0.0002 | 0.0003 | 0.0004 | 0.0008 | 0.0012 | 0.0022 | 0.0004 | 0.0004 | 0.0003 | 0.0002 | 0.0007 | 0.0005 | 0.0005 | 0.0007 | 0.0004 | 0.0007 |
